# A novel isolation method for spontaneously released extracellular vesicles from brain tissue and its implications for stress-driven brain pathology

**DOI:** 10.1186/s12964-023-01045-z

**Published:** 2023-02-13

**Authors:** Patrícia A. Gomes, Cristian Bodo, Carlos Nogueras-Ortiz, Martina Samiotaki, Minghao Chen, Carina Soares-Cunha, Joana M. Silva, Bárbara Coimbra, George Stamatakis, Liliana Santos, George Panayotou, Foteini Tzouanou, Clarissa L. Waites, Christos Gatsogiannis, Nuno Sousa, Dimitrios Kapogiannis, Bruno Costa-Silva, Ioannis Sotiropoulos

**Affiliations:** 1 Life and Health Sciences Research Institute (ICVS), School of Medicine, University of Minho, Campus de Gualtar, 4710-057 Braga, Portugal; 2 ICVS/3B’s - PT Government Associate Laboratory, Braga/Guimarães, Portugal; 3 Systems Oncology Group, Champalimaud Research, Champalimaud Centre for the Unknown, Av. Brasília, 1400-038 Lisbon, Portugal; 4 Laboratory of Clinical Investigation, Intramural Research Program, National Institute on Aging, NIH, Baltimore, MD USA; 5 Institute for Bioinnovation, Biomedical Sciences Research Center “Alexander Fleming”, 16672 Vari, Attica, Greece; 6 Center for Soft Nanoscience and Institute of Medical Physics and Biophysics, University of Muenster, 48149 Münster, Germany; 7 Department of Structural Biochemistry, Max Planck Institute of Molecular Physiology, 44227 Dortmund, Germany; 8 Institute of Biosciences and Applications NCSR “Demokritos”, Athens, Greece; 9 Department of Pathology and Cell Biology, Taub Institute for Research on Alzheimer’s Disease and the Aging Brain, Columbia University Medical Center, New York, NY USA

**Keywords:** Extracellular vesicles, Brain, Exosomes, Human, Mouse, Spontaneous release, Stress

## Abstract

**Background:**

Extracellular vesicles (EVs), including small EVs (sEVs) such as exosomes, exhibit great potential for the diagnosis and treatment of brain disorders, representing a valuable tool for precision medicine. The latter demands high-quality human biospecimens, especially in complex disorders in which pathological and specimen heterogeneity, as well as diverse individual clinical profile, often complicate the development of precision therapeutic schemes and patient-tailored treatments. Thus, the collection and characterization of physiologically relevant sEVs are of the utmost importance. However, standard brain EV isolation approaches rely on tissue dissociation, which can contaminate EV fractions with intracellular vesicles.

**Methods:**

Based on multiscale analytical platforms such as cryo-EM, label-free proteomics, advanced flow cytometry, and ExoView analyses, we compared and characterized the EV fraction isolated with this novel method with a classical digestion-based EV isolation procedure. Moreover, EV biogenesis was pharmacologically manipulated with either GW4869 or picrotoxin to assess the validity of the spontaneous-release method, while the injection of labelled-EVs into the mouse brain further supported the integrity of the isolated vesicles.

**Results:**

We hereby present an efficient purification method that captures a sEV-enriched population spontaneously released by mouse and human brain tissue. In addition, we tested the significance of the release method under conditions where biogenesis/secretion of sEVs was pharmacologically manipulated, as well as under animals’ exposure to chronic stress, a clinically relevant precipitant of brain pathologies, such as depression and Alzheimer’s disease. Our findings show that the released method monitors the drug-evoked inhibition or enhancement of sEVs secretion while chronic stress induces the secretion of brain exosomes accompanied by memory loss and mood deficits suggesting a potential role of sEVs in the brain response to stress and related stress-driven brain pathology.

**Conclusions:**

Overall, the spontaneous release method of sEV yield may contribute to the characterization and biomarker profile of physiologically relevant brain-derived sEVs in brain function and pathology.

**Video Abstract**

**Supplementary Information:**

The online version contains supplementary material available at 10.1186/s12964-023-01045-z.

## Introduction

The advent of Precision medicine demands high-quality biomarkers, especially for complex brain disorders, where pathological heterogeneity and diverse clinical presentations complicate the development of patient-tailored and disease-modifying treatments. Therefore, it is important to consider how these biomarkers are harvested. In the past, free proteins detectable in the blood and cerebrospinal fluid (CSF) contributed to disease diagnosis; however, their bioavailability is extremely low, representing less than one millionth of the total proteins available in these biofluids [1]. Similarly, amplified free nucleic acids present a low signal-to-noise ratio that impedes their use as biomarkers [2]. Hence, biomarker discovery has been shifted to other biofluid constituents, such as nano-sized extracellular vesicles (EVs).

**Fig. 1:**
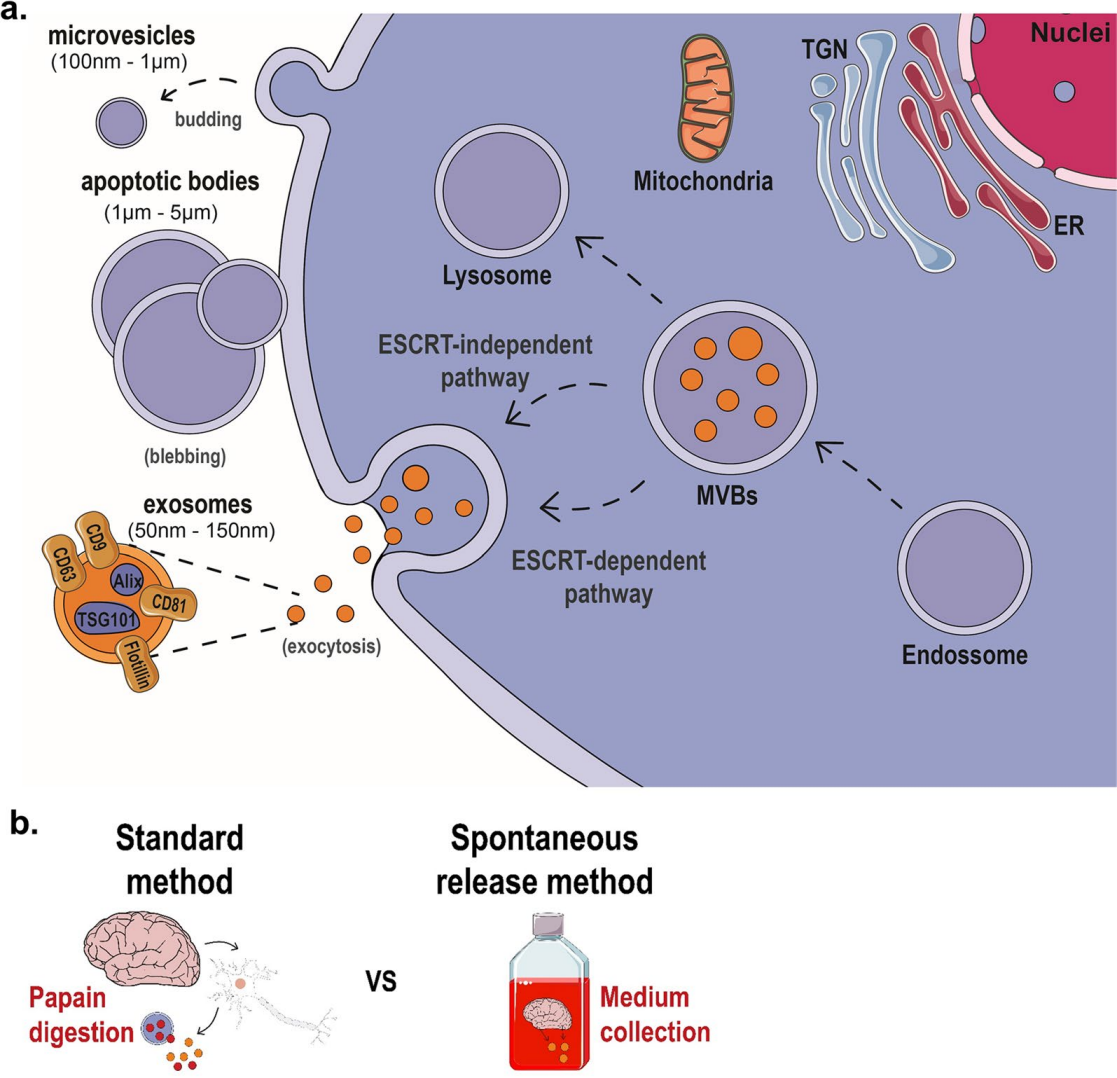
Biogenesis and isolation of extracellular vesicles from brain tissue. **a** Schematic representation of the biogenesis of three types of extracellular vesicles (EVs): microvesicles, apoptotic bodies, and exosomes. On the one hand, microvesicles arise from the budding of the plasma membrane, while apoptotic bodies originate from cell blebbing. On the other hand, exosomes are released upon multivesicular bodies (MVB) fusion with the plasma membrane. MVBs develop following invagination of early endosomes with intraluminal vesicles (ILVs). These ILVs can be degraded upon fusion with lysosomes or released as exosomes. MVB-derived exosome biogenesis can involve the ESCRT machinery or occur via an ESCRT-independent pathway. **b** Schematic representation of the main idea of the 2 methods compared in the current studies: the standard method which includes chemical and mechanical (e.g., papain) digestion of the brain tissue and the release method for brain sEVs collection, which isolates EVs that are spontaneously released by the brain tissue

EVs, including endosomal-origin exosomes (50–150 nm) and larger microvesicles originating from the plasma membrane (Fig. 1a), are important mediators of intercellular and inter-organ communication [3], contributing to neuronal development and function [4, 5]. These bilipid membranous structures protect their cargo, including proteins and microRNA, from the extracellular milieu. They can cross the blood–brain barrier to the periphery [6, 7], where they can be harvested for their biomarker carrier capacity. Due to their intracellular origin, cargo protecting-abilities, and long half-lives, brain EVs reflect molecular processes in their originating cells, exhibiting great potential in diagnostics, treatment follow-up, and therapeutics for diverse neurological, neurodegenerative and psychiatric disorders, including brain cancer, multiple sclerosis, Alzheimer’s and Parkinson’s disease, major depression and schizophrenia [2, 8, 9]. Therefore, the collection of pure and physiologically relevant brain EVs, which constitute the gold-standard population against which peripheral EV biomarkers ought to be compared, is of the utmost importance. Nevertheless, the efficient, accurate, and selective isolation, identification, and quantification of brain EVs remain a challenge.

Standard methods for brain EV isolation [10–12] rely on tissue dissociation, which may lead to cellular damage and disruption, contaminating the EV fraction with intracellular components, e.g., immature intraluminal vesicles (ILVs) within late endosomes that would otherwise be targeted for degradation instead of exocytosis (Fig. 1a) [12]. Any such contamination may lead to the inaccurate characterization of brain EVs profile and further delay the discovery of disease-relevant biomarkers. Considering this, we propose a novel method to isolate small EVs (sEVs) upon their release from fresh brain tissue into culture media—cell’s physiological process of sEVs biogenesis. sEVs are then isolated to obtain a highly pure brain EVs fraction that we have rigorously and extensively characterized. Importantly, we have also demonstrated that the isolated EVs are functional as they are internalized by neurons upon injection in the brain. Moreover, using this new method, we studied the pharmacologically- or neuroactivation-induced modulation of EVs release ex vivo*,* as well as under chronic stress, a clinically relevant precipitant of brain pathologies such as depression and Alzheimer’s disease. Therefore, by overcoming the drawbacks of the standard, currently used protocols, this novel method opens new avenues for the study of EVs of the nervous system in health and disease, constituting a clinically relevant tool for the detailed profile of physiologically relevant EVs released directly from brain cells.

## Material and methods

### Animal and human samples

Two- to four-month-old WT animals (C57BL/6J) were used in this study. Mice were housed in groups of 5–6 per cage under standard environmental conditions (lights on from 8 a.m. to 8 p.m.; room temperature 22 °C; relative humidity of 55%, ad libitum access to food and water). For chronic stress experiments, animals were subjected to chronic unpredictable stress protocol over a period of 6 weeks before the behavioral testing. As previously described [13], the stress protocol consists of different stressors, such as overcrowding (3 h), rocking platform (3 h), restrain (3 h), and hairdryer (30 min), (one stressor per day) that were chosen randomly to prevent habituation. Brain tissue was collected 1 day after the last behavioral test. For human brain samples analysis, the anterior frontal cortex of 3 individuals (2 females and 1 male, 46–78 years, post-mortem delay 9–28 h) was obtained from the Portuguese Brain Bank. In each case, 300 mg of frontal cortex tissue was flash-frozen for isolation of small EVs (sEVs), while another 300 mg piece of the same tissue was cultured as described below.

### Animal behavioral testing

For monitoring memory performance, Contextual Fear Conditioning was conducted in chambers (20 cm × 16 cm × 20.5 cm; Med Associates, St. Albans, VT) within an insulated white plastic cabinet that provided protection from outside light and noise. A light (CM1820 bulb) mounted directly above the chamber provided illumination. On day 1, mice were placed in the white conditioning chamber (Context A) and received three pairings of light (20 s) which co-terminated with an electrical shock (2 s, 0.5 mA). On day 2, animals were again placed in context A (familiar context) in the absence of the light-shock pairings. After testing, the animals returned to their home cage. The chambers were cleaned with 10% ethanol between animal trials. Mice behavior was recorded by a video camera, and freezing behavior was measured manually for 3 min using Kinoscope software as previously described [14]. For depressive-like behavior, we performed tail suspension test (TST) where mice were suspended by the tail for 5 min; the immobility time of the animal was manually measured with Kinoscope software.

### GW4869 and picrotoxin treatment

For GW4869 and picrotoxin experiments, mouse brain was incubated with GW4869 (cat. No. D1692, Sigma-Aldrich) which was dissolved in tissue culture–grade DMSO at 0.2 mg ml^−1^and added to the EV-release medium to a final concentration of 20 µM. Picrotoxin (cat. No. 1128/1G, Tocris) was dissolved in tissue culture–grade DMSO at 100 mM. Picrotoxin solution was added to the EV-release medium to a final concentration of 100 µM. After brain tissue incubation for 16 h in the EV-release medium supplemented with GW4869 or Picrotoxin, EVs were isolated and analyzed as mentioned above. The concentration of each drug was chosen based on previous brain studies conducted in cell lines or brain slices, avoiding toxicity [15, 16].

### Isolation of brain extracellular vesicles

For the standard method [10, 12], five hemi-cortices were pooled into one cortical sample, while the hippocampi and entorhinal cortices of five mice were pooled to obtain one hippocampal and entorhinal sample, respectively. Animals were sacrificed, brain tissue collected, and snap-frozen. Minced cortical brain tissue was incubated for 20 min at 37 °C with previously activated papain (Worthington, LK003178; 20units/mL, 37 °C for 10 min) in Hibernate-A (Gibco™, A1247501). After the addition of cold hibernate-A with protease and phosphatase inhibitors [50X cOmplete™, EDTA-free Protease Inhibitor Cocktail (Sigma, #11873580001); phosphatase inhibitor cocktail 2 (Sigma, #P5726) and 3 (Sigma, #P0044)], tissue was loosened up and centrifuged at 300 g (10 min, 4 °C). Following 40 µm-mesh filtering, the supernatant was centrifuged at 2.000 g (10 min, 4 °C) and then at 10.000 g (30 min, 4 °C). The resulting supernatant was filtered through a 0.2 µm syringe filter and centrifuged at 100.000 g (70 min, 4 °C). Following pellet resuspension and centrifugation at 100.000 g (70 min, 4 °C), this second pellet was resuspended in 40 w/v % OptiPrep™ (D1556, Sigma-Aldrich) solution and layered on the bottom of a new tube. Then, after layering a discontinuous OptiPrep gradient (40 w/v %, 20 w/v %, 10 w/v %, and 5 w/v % OptiPrep solution), this gradient column was centrifuged at 200.000 g (16 h, 4 °C). Then, the desired fractions were collected, i.e., layers 4–9 corresponding to small extracellular vesicles. After adding ice-cold PBS to each fraction, these were centrifuged at 100.000 g (70 min, 4 °C), and each pellet was collected. For the release method, animals were sacrificed, brain tissue was immediately macrodissected and incubated in EV-release medium (Neurobasal, 1%Glutamax, 1%Anti-anti; Gibco™, 21103049, 35050038, 15240062, respectively) for 16 h at 37 °C, 5% CO_2_. Specifically, five hemi-cortices were pooled to obtain one cortical EV sample while hippocampi and entorhinal cortices from five mouse brains were pooled into hippocampal and entorhinal EV samples, respectively. After sequential centrifugation of the conditioned EV-release medium to remove cell fragments, apoptotic bodies, and larger microvesicles (500 g, 10 min; 3000 g, 20 min; 12.000 g, 20 min; 10 °C), the last supernatant was filtered through a 0.2 µm syringe filter and centrifuged at 100.000 g to acquire small EVs (70 min, 10 °C). After pellet resuspension, the suspension was loaded onto a qEV column (IZON©, SP1) and the previously described protocol for small EVs collection was used [17]. The desired small EV fraction (1.5 mL) was mixed with cold PBS (ratio 1:10) and this solution was layered on top of 4 mL of a sucrose cushion (D2O containing 1.2 g of protease-free sucrose and 96 mg of Tris base, pH 7.4, 0.2 µm filtered) and centrifuged at 100.000 g (70 min, 10 °C). Lastly, 4 mL from the bottom of the tube were collected with an 18G syringe and diluted in 16 ml of PBS before being centrifuged at 100.000 g (16 h, 10 °C). Finally, the pellet containing EVs was collected and stored for further use and analysis.

### Cryogenic electron microscopy (Cryo-EM)

To visualize the isolated vesicles and assess their purity status, 4 µl of undiluted EV samples were applied to glow-discharged holey carbon grids with an additional carbon layer (R2/1 300 Mesh + 2 nm C, QUANTIFOIL and incubated for 5 min before blotting. The grids for cryo-EM analysis were prepared with Vitrobot (Thermo Fisher) at 4℃ with 100% humidity. The sample application was repeated two times to increase the concentration of sEVs. After final blotting, the grids are plunge-frozen in liquid ethane and stored in liquid nitrogen. Images were collected with Talos Arctica (Thermo Fisher) equipped with a Falcon 3 direct electron camera (Thermo Fisher) at an acceleration voltage of 200 kV and a magnification of 120,000 (pixel size of 1.21 Å on the specimen level). Images were collected at a fixed defocus value of -3.0 um for release and standard methods, respectively. From the acquired images, the diameter of 123 and 144 sEVs were measured manually for release and standard methods, respectively, using FIJI [18]. For the elliptic sEVs, the average lengths of the long and short axis were used.

### Nanoparticle tracking analysis

All EV samples were analyzed by NS300 Nanoparticle Tracking Analysis (NTA) system with a red laser (638 nm) (Malvern Panalytical, UK). Samples were diluted in filtered PBS to achieve a concentration within the range for optimal NTA analysis. Video acquisitions were performed using a camera level of 16 and a threshold of 5. Five videos of 30 s were captured per sample. Analysis of particle concentration per mL and size distribution were performed with the NTA software v3.4 (Malvern Panalytical, Malvern, UK).

### Proteomic analysis of EVs

#### Sample preparation using Sp3-mediated protein digestion

The lysates of the equivalent to 4 × 10^8^ purified sEV were processed according to the sensitive Sp3 protocol [19]. The cysteine residues were reduced in 100 mM DTT and alkylated in 100 mM iodoacetamide (Acros Organics). 20 ug of beads (1:1 mixture of hydrophilic and hydrophobic SeraMag carboxylate-modified beads, GE Life Sciences) were added to each sample in 50% ethanol. Protein clean-up was performed on a magnetic rack. The beads were washed two times with 80% ethanol and once with 100% acetonitrile (Fisher Chemical). The captured beads proteins were digested overnight at 37 °C under vigorous shaking (1200 rpm, Eppendorf Thermomixer) with 0.5 ug Trypsin/LysC (MS grade, Promega) prepared in 25 mM Ammonium bicarbonate. Next day, the supernatants were collected and the peptides were purified using a modified Sp3 clean-up protocol and finally solubilized in the mobile phase A (0.1% Formic acid in water), sonicated, and the peptide concentration was determined through absorbance at 280 nm measurement using a nanodrop instrument.

#### LC–MS/MS analysis

Samples were run on a liquid chromatography tandem mass spectrometry (LC–MS/MS) setup consisting of a Dionex Ultimate 3000 nano RSLC online with a Thermo Q Exactive HF-X Orbitrap mass spectrometer. Peptidic samples were directly injected and separated on a 25 cm-long analytical C18 column (PepSep, 1.9 μm^3^ beads, 75 µm ID) using a 1-hour long run, starting with a gradient of 7% Buffer B (0.1% Formic acid in 80% Acetonitrile) to 35% for 40 min and followed by an increase to 45% in 5 min and a second increase to 99% in 0.5 min and then kept constant for equilibration for 14.5 min. A full MS was acquired in profile mode using a Q Exactive HF-X Hybrid Quadropole-Orbitrap mass spectrometer, operating in the scan range of 375–1400 m/z using 120 K resolving power with an AGC of 3 × 106 and max IT of 60 ms followed by data independent analysis using 8 Th windows (39 loop counts) with 15 K resolving power with an AGC of 3 × 105 and max IT of 22 ms and normalized collision energy (NCE) of 26.

#### Data analysis

Orbitrap raw data was analyzed in DIA-NN 1.8 (Data-Independent Acquisition by Neural Networks) [20] through searching against the reviewed Mus musculus Uniprot database (retrieved 4/21) in the library free mode of the software, allowing up to two tryptic missed cleavages. A spectral library was created from the DIA runs and used to reanalyze them. DIA-NN default settings have been used with oxidation of methionine residues and acetylation of the protein N-termini set as variable modifications and carbamidomethylation of cysteine residues as fixed modification. N-terminal methionine excision was also enabled. The match between runs feature was used for all analyses and the output (precursor) was filtered at 0.01 FDR and finally, the protein inference was performed on the level of genes using only proteotypic peptides. The generated results were processed statistically and visualized in the Perseus software (1.6.15.0) [21]. Potential contaminants, decoy proteins, and proteins only identified by one site were filtered out. Finally, we used ConsensusPathDB [22, 23] and DAVID [24, 25] to detect significant cellular component GO categories for the abundant proteins found previously. The mass spectrometry proteomics data has been deposited to the ProteomeXchange Consortium via PRIDE [26] partner repository with the dataset identifier PXD031947.

### Western blot analysis

Western blot analysis of mouse brain EV samples was performed using RIPA buffer (50 mM Tris–HCl, 2 mM EDTA, 250 mM NaCl, 10% glycerol); RIPA was added to 5 × 10^9^ EVs followed by Laemmli buffer, and samples were boiled at 98 °C for 10 min. Samples were loaded onto pre-cast gels (4–20% Mini-PROTEAN® TGX Stain-Free™ Protein Gels, 10 well, 50 µl, cat.no. 4568094, Biorad) and semi-dry transferred onto nitrocellulose membranes (Trans-Blot Turbo blotting system, BIORAD). Membranes were blocked with 5% non-fat dry milk in TBS-T buffer and then incubated with the following antibodies in 2.5% non-fat dry milk in TBS-T buffer: cytochrome C (1:1000, ab13575, ABCAM), CD81 (1:400, sc-166029, Santa Cruz), flotillin-1 (1:500, sc-133153, Santa Cruz), EAA-1 (1:1000, #3288, Cell Signaling). After overnight incubation with primary antibodies (4 °C), membranes were incubated with appropriate secondary antibody for 2 h at RT (1:10000; IRDye 680RD Goat anti-Rabbit #926-68071; IRDye 680RD Donkey anti-Mouse #926-68072; IRDye 800CW Goat anti-Mouse #926-32210; IRDye 800CW Goat anti-Rabbit #926-32213). The antigen signal was revealed using Sapphire Biomolecular Imager (Azure Biosystems, Dublin, CA) and AzureSpot software (Azure Biosystems, Dublin, CA).

### Flow cytometry detection of EVs

EV samples of ~ 30 to 40 µL volume were diluted to a final volume of 1.25 mL using 1X-PBS supplemented with 0.05% tween-20 detergent and split in five 250 µL aliquots which were subjected to labeling with the following fluorescent-tagged anti-mouse antibodies at 0.2 ng/µL and 4 °C overnight: rat IgG2a anti-CD9 (Biolegend, cat. no. 124802), rat IgG2a anti-CD63 (Biolegend, cat. no. 143901) and Armenian hamster IgG anti-CD81 (Biolegend, cat. no. 104901), using rat IgG2a κ (Biolegend, cat. no. 400501) and Armenian hamster IgG (Biolegend, cat. no. 400901) isotype control antibodies (BioLegend, San Diego, CA). The water and PBS used for antibody and sample dilution was filtered using a 20 nm inorganic membrane filter (cat. no. 6809–1002; Whatman, Hillsboro, OR) to remove nanoparticles that could contribute to noise to the flow cytometry analysis. A set of EVs that were treated with 1% NP40 detergent (Sigma-Aldrich, St. Louis, MO) and sonicated for 1 min as well as another set of EVs that were isolated from cpVenus-expressing HEK cells via differential ultracentrifugation as previously described [27] were used as controls. Labeled EVs were detected with a CytoFLEX LX flow cytometer (Beckman Coulter, Indianapolis, IN) using 405-SSC triggering and analyzed with CytExpert software v2.3.0.84 (Beckman Coulter). For fluorescent detection, we used a 525/40 bandpass filter for FITC, 660/10 for APC, and 585/42 for PE, with gain voltage not exceeding 1500 V. The instrument was aligned using FITC-tagged beads with sizes ranging from 100 to 1300 nm (100 nm beads, cat. no. 834, Bangs Laboratories, Fishers, IN; 130–1300 nm beads, cat. no. NFPPS-52-4K and NFPPS-0152-5, Spherotech, Libertyville, IL). Samples were diluted with 1X-PBS supplemented with 0.05% tween-20 to control the abort rate below 1% without exceeding 100,000 events/second rate to avoid coincident detection of events and analyzed until reaching 500,000 events.

### Extracellular vesicles labeling and stereotaxic injection in mouse brain

During the EVs isolation process, and before qEV column use, mouse brain EVs were labeled using 5 µM DiI (cat. No. D282, Invitrogen/Molecular Probes) for 30 min at 4 °C (gentle rotation). Then, the isolation protocol was concluded to obtain a pellet of DiI-labelled sEVs. DiI-labeled EVs were quantified by NTA and BCA as described above. Animals were stereotaxically injected with DiI-labeled EVs and corresponding controls (see below) into the outer molecular layer (OML) of the dentate gyrus (DG) with the following brain coordinates: anteroposterior, − 2.18 mm; lateral, 1.25; dorsoventral, − 1.60. The different control groups included injection of equal volumes of PBS, DiI dye alone, or DiI dye alone which was processed through the release protocol. Animals were sacrificed 1 day and 4 weeks after the injection as previously described [28]. Briefly, deeply anesthetized animals were transcardially perfused with saline and PFA (4%). After post-fixation for 24 h at RT in 4% PFA, brains were placed in 30% sucrose and sectioned using vibrating-blade microtome (VT1000S, Leica, Germany). 30 µm slices were incubated for 10 min, room temperature, with DAPI (1 mg/ml) and mounted using mounting media (Permafluor, Invitrogen, MA, USA). Images were collected by confocal microscopy (Olympus FluoViewTMFV3000, Olympus, Tokyo, Japan).

### ExoView analysis

EVs samples were analyzed using the ExoView Mouse Tetraspanin Kit (NanoView Biosciences, USA). For tetraspanin detection, samples were diluted at 1:12,000 prior to overnight incubation. Chips were washed three times in solution A and then incubated with a cocktail containing anti-CD9 CF488, anti-CD81 CF555, and anti-CD63 CF647. All fluorescent antibodies were diluted as per the manufacturer’s instructions (1:1000 for tetraspanins). After 1-h incubation at room temperature, chips were washed in kit-supplied buffers, dried, and imaged by the ExoView R100 using nScan v2.9.5. Data was analyzed using NanoViewer 2.9.5. Fluorescent cut-offs were set relative to the MIgG control.

### Statistical analysis

Statistical analysis was performed with GraphPad Prism v8 (GraphPad Software, La Jolla, USA) using Welsh t-test to compare group means after normality assessment by Shapiro-Wilks statistical test. If normality was not verified, the Mann–Whitney test was used to compare group ranks. Values were considered as significant when *p* < 0.05, and all the results were expressed as mean ± SEM (standard error of mean).

## Results

### The release method isolates a homogenous small EVs population

**Fig. 2:**
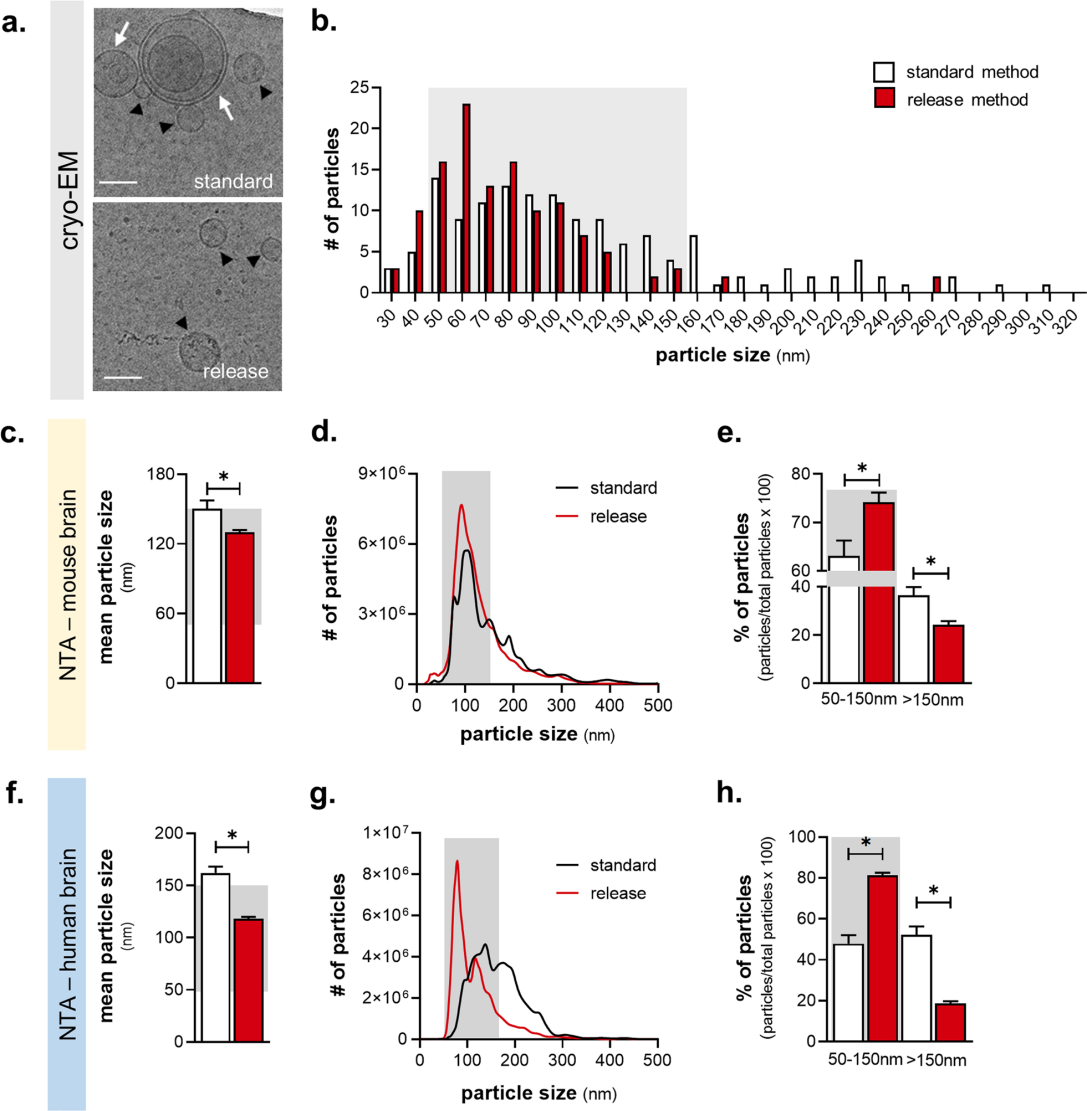
The release method provides higher purity small EV yield. **a**, **b** Cryo-EM micrographs (**a)** of isolated EVs (black arrowheads; multi-lamellar highlighted by white arrows; scale bar = 100 nm) and their size distribution (**b)** with grey region highlighting the size area of small EVs [50–150 nm]) (vesicles: N_*standard*_ = 123, N_*release*_ = 144). **c–h** Nanoparticle tracking analysis (NTA) of the mean size (**c**, **f**) and size distribution (**d**, **g**) of particles from mouse and human cortex, respectively. Small EVs data are highlighted in the grey area (**e**, **h**) (mouse: N_*standard*_ = 7, N_*release*_ = 8; human: N_*standard*_ = 3, N_*release*_ = 3). All data shown represent mean ± SEM *p < 0.05 by two-tailed Welch’s t-test

We developed a novel method to isolate small EVs (sEVs) from the brain, relying on their spontaneous release (referred to as the ‘release method’) to overcome the potential contamination by cellular disruption when chemical and mechanical dissociation is used. Dissected brain tissue was incubated in culture medium followed by EV isolation with ultracentrifugation, size-exclusion chromatography (SEC), and density separation (Fig. 1b). Then, we compared the release method to a standard method for isolation of brain EVs [10, 12] using both mouse and human cortical tissue and the resulting EVs were characterized according to MISEV2018 guidelines [29]. Based on cryo-EM imaging and size distribution analysis (Fig. 2a, b), we found that the release method isolated a more homogeneous population of smaller EVs of the mouse brain, as well as a larger number of smaller EVs (50–150 nm) compared to the standard method. Importantly, EVs isolated by the standard method included multi-lamellar vesicles (Fig. 2a, Additional file 1: Fig. S1a), which were not detected in the EVs samples of the release method. Next, nanoparticle tracking analysis (NTA) confirmed that the mean diameter of isolated EVs of the release method was smaller compared to the standard one (Fig. 2c; t-test with Welch’s correction, t(14) = 2.788, *p* = 0.027) showing a clear enrichment of smaller EVs (50–150 nm) (Fig. 2d, e; Mann–Whitney, U_50–150 nm_ = 9, *p* = 0.026; U_>150 nm_ = 7, *p* = 0.014). The purity of EV yield for the two methods was further compared based on the correlation between the number of particles and the total protein of the EV fraction measured by NTA and microBCA, respectively [29]. Despite the release protocol providing a smaller yield of EV total protein, it displayed a stronger protein:particle correlation compared to the standard method (Additional file 1: Fig. S1b; Pearson r, r_standard_(14) = 0.1406, *p* = 0.407; r_release_(14) = 0.883, *p* < 0.001), suggesting higher EV purity. A similar EV profile of the release method was found when human cortical tissue was used (Fig. 2f–h; mean size: t-test with Welch’s correction, t(4) = 3.135, *p* = 0.05, t(4) = 6.999, *p* = 0.012; particle distribution: t-test with Welch’s correction, t_50–150 nm_(4) = 7.785, *p* = 0.01, t_>150 nm_(4) = 7.826, *p* = 0.01). Additionally, we confirmed the efficiency of the release method in smaller brain areas of mouse brain such as the entorhinal cortex and hippocampus; these brain areas are among the first to be affected in brain disorders such as Alzheimer’s disease as well as stress-related brain disorders, e.g., depression [30, 31]. Hippocampal and entorhinal cortex EVs exhibited a similar size profile compared to cortical EVs, although lower concentrations of EV particles and protein were recovered in entorhinal and hippocampal tissue, indicating a wide range of workable tissue weights and brain areas for the release method (Additional file 1: Fig. S2a-c). In addition, tissue weight influenced the total number of particles, but not their size distribution (Additional file 1: Fig. S2d–e; Pearson r, r_size_(16) = 0.1147, *p* = 0.6723; r_particles_(15) = 0.8588, *p* < 0.0001) further supporting the reliability of the release method. Finally, we also performed quality control of the brain tissue at the end of the incubation period (16 h) of the release method comparing it to 0 and 40 h of incubation as well as H_2_O_2_ treatment (Additional file 1: Fig. S3 a,b). In contrast to the significant reduction of the number of cells and % of alive cells after 40 h incubation or H_2_O_2_ treatment, the release method incubation period did not exhibit a significant effect on cell survival [F_cells_(3,8) = 10.07, *p* = 0.0043; Tukey’s post-hoc: *p* = 0.9997 or *p* < 0.05; F_alive_(3,8) = 23.65, *p* = 0.0002; Tukey’s post-hoc: *p* = 0.4147 or *p* < 0.05]. In line with these findings, WB analysis demonstrated no effect of the release method on protein levels of Cytochrome C (CytC) and Neuron-specific enolase (NSE)—both associated with cell damage and/or death—whereas there was a significant impact of 40 h incubation and/or H_2_O_2_ treatment [Additional file 1: Fig. S3c-e; F_CytC_(3,32) = 8.844, *p* = 0.0002; Tukey’s post-hoc: *p* < 0.05; F_NSE_(3,32) = 3.585, *p* = 0.0243; Tukey’s post-hoc: *p* < 0.05].

### The EV fraction of the release method is enriched in proteins common to extracellular vesicles

**Fig. 3:**
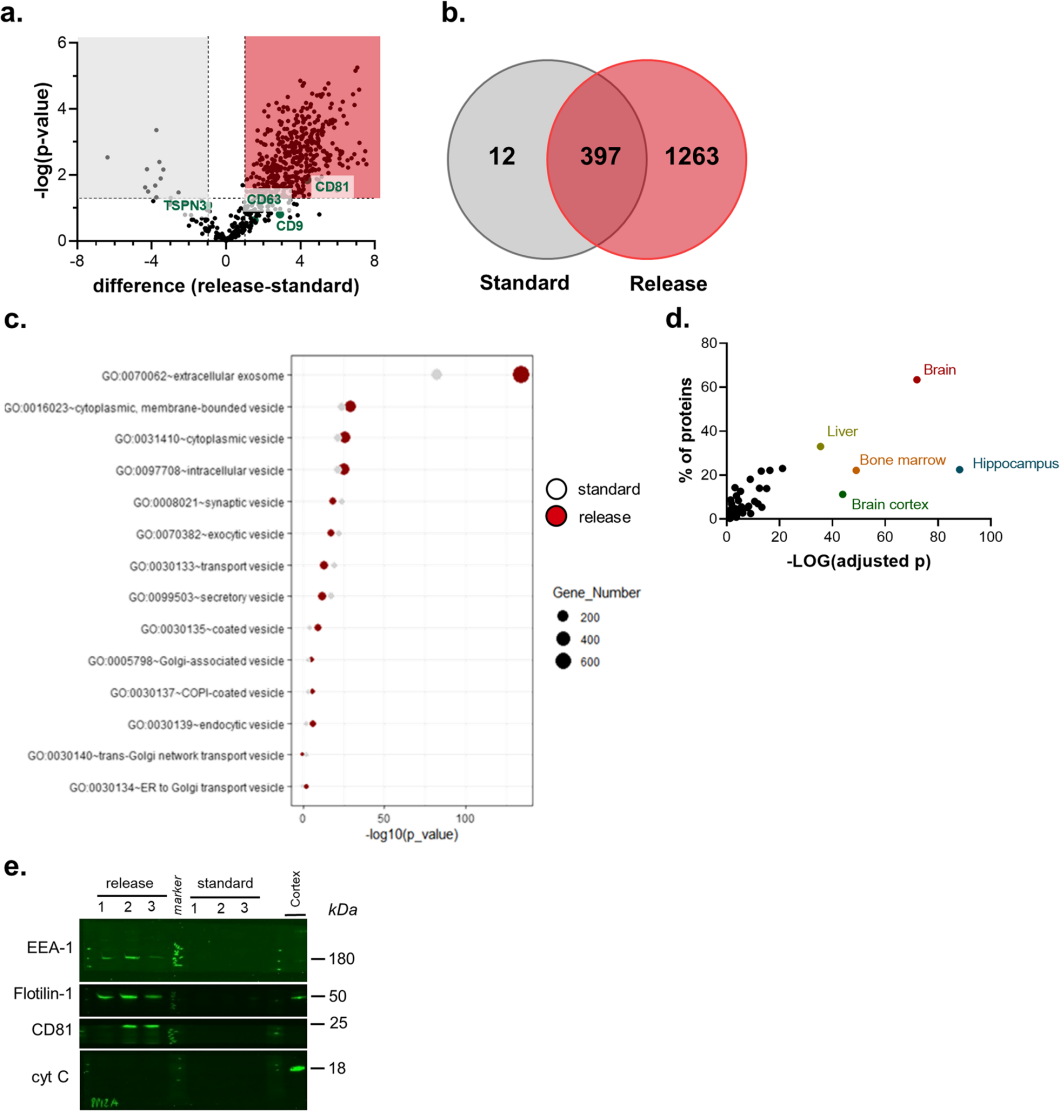
The release method enriches for EV-associated proteins. **a** Volcano plot of the detected proteins of the GO category “extracellular exosome”. **b** Venn diagram of the detected proteins between release and standard method. **c** Cellular component analysis of EVs proteome exhibiting higher enrichment of categories related to small EVs (e.g., exosomes) in the release method (based on ConsensusPathDB). **d** Tissue derivation of EV proteins of the release method (based on DAVID knowledgebase), showing enrichment in brain- and hippocampus-specific proteins. **e** Representative immunoblots of mouse brain EVs from the standard and release methods, showing the presence of EV-related proteins (e.g., CD81, flotillin) and endosomal proteins, (e.g., EEA-1), but not the mitochondrial protein cytochrome c (N = 3 per condition)

To monitor the proteomic profile of EVs isolated by both methods, we next performed label-free proteomic analysis of similar EVs number. As shown by the Volcano plot and Venn diagram (Fig. 3a, b), our analysis revealed a higher number of proteins in the EVs yield of the release protocol (1660 proteins) compared to the standard one (409 proteins) with some classical EVs markers enriched in the EVs of the released method, e.g., CD9, CD81 (Fig. 3a). Among the detected proteins, 397 proteins were commonly detected between the 2 protocols while 1263 versus 12 proteins were detected only in the release versus the standard method (Fig. 3b). Moreover, as shown in Fig. 3c, the proteomic analysis revealed that, in contrast to the standard method, the EV yield isolated by the release method was enriched in proteins of the cellular component category “extracellular exosome” (GO: 0070062) whereas the rest of the detected GO categories appeared similar between the release and standard method, supporting further the findings of electron microscopy (EM) and nanoparticle tracking analysis (NTC) which indicates an exosome-enriched yield obtained by the release method (see also Fig. 2). Furthermore, as shown in Fig. 3d and Table 1, the vast majority are brain-related and neuronal-related proteins with EV markers, cytosolic proteins, annexins, RABs, and cytoskeleton proteins among others. Regarding the proteins that are exclusively present in the release protocol, note that the GO categories with high FDR belongs to the extracellular region categories exhibiting approx. 40% relative enrichment within the EV yield of the release method (Additional file 1: Fig. S4). In addition, proteins of GO category “synaptic part” also exhibit approx. 15% relative enrichment; a finding that is in agreement with previous studies supporting the presence of synaptic proteins in the exosome cargo and their potential use as biomarkers in disorders characterized by synaptic malfunction/loss e.g. Alzheimer’s disease [9]. Western blot analysis of the EVs yield of the two methods confirmed the proteomic findings by detecting a higher abundance of EV markers (i.e., CD81, flotillin-1) and proteins such as EEA1, which are derived from the endolysosomal pathway, the origin of sEVs such as exosomes, in the release protocol (Fig. 3e).

**Table 1:** CNS cell markers detected by proteomic analysis of the release method EVs divided into four cell-origin categories (neurons, microglia, astrocytes, oligodendrocytes)

*CNS cell markers*
Abcb1a, Acadl, Acp1, Add2, Agap2, Agpat3, Ahcyl2, Aifm3, Ak1, Alcam, Aldh5a1, Aldoc, Amph, Ank3, Anks1b, Anxa5, Arhgef9, Arl8a, Arpc5, Ass1, Atp1a3, Atp1b1, Atp2b4, Atp6v1g2, Atxn2, Bcan, Bcat1, Bin1, Brsk2, Cacna1e, Cacna2d1, Camk2a, Camk2b, Camk2g, Cap2, Capn2, Capn5, Carhsp1, Cat, Cd200, Cd47, Cd63, Cdk14, Cds1, Cept1, Chmp1a, Chordc1, Cit, Ckmt1, Clec2l, Clu, Cmtm4, Cnksr2, Cntnap1, Copb2, Cotl1, Cpne5, Cpne6, Crbn, Crtac1, Cryab, Cs, Cse1l, Csrp1, Ctbp1, Cul3, Cx3cl1, Cyfip2, Cyp46a1, Dbn1, Dclk1, Dclk2, Ddah1, Ddx1, Dhrs1, Diras1, Diras2, Dkc1, Dlat, Dlgap2, Dmd, Dpysl3, Dynll2, Edil3, Eif2b4, Bem, Endod1, Eno1, Epb41l1, Epb41l3, Erlin2, Exoc5, Ezr, Faah, Fam234b, Fbxl16, Fbxo41, Fkbp5, Fn3k, Gabrb2, Gabrg2, Galk2, Gap43, Gart, Gdpd1, GFAP, Glul, Gmppa, Gmpr, Gnao1, Gnaz, Gnb4, Gng2, Gng7, Golga7b, Gpd1l, Gpr37, Gria3, Gria4, Grin1, Gstp1, Hapln1, Hdac2, Hdlbp, Hpca, Hpcal4, Hspa4l, Hspb1, Hsph1, Igsf8, Inpp5f, Iqsec2, Itgb1, Itsn1, Kcnab2, Kcnk1, Kctd12, Kif3a, Ldha, Lin7a, Lmna, Lpar1, Lsamp, Ly6h, Lynx1, Map1a, Map1b, MAP2, Map6, Map7d1, Mapk3, Mapre3, Marcks, Mboat2, Mcts1, Me1, Mif, Mpdu1, Mpp6, Mtap, Myh10, Nae1, Nat8l, Ncald, Ncam1, Ncdn, Nckap1, Nckipsd, Nefl, Nme3, Nop56, Nrgn, Ntm, Ocrl, Ola1, Osbpl1a, Pacsin1, Pafah1b1, Pfkfb2, Pfkm, Pfkp, Pgbd5, Pgd, Pgm2l1, Phf24, Phyhip, Pik3r4, Pip4k2a, Pitpna, Pitpnc1, Plcb1, Plcl1, Plxna4, Por, Ppm1e, Ppp1r7, Ppp2r5e, Prdx1, Prex1, Prkar2b, Prkcb, Prkcd, Prpf19, Prps1, Prrt2, Prrt3, Ptdss2, Pten, Ptgr2, Ptpn5, Pygm, Rab3c, Rab6b, Rala, Rap1a, Rap1b, Rap2a, Rcc2, Rdh14, Reep2, Reep5, Rgs6, Rps18, Rps6, Rrbp1, Rtn1, Ryr2, S100a13, Scarb2, Scn1b, Scn2b, Scn4b, Sec31a, Sec61a1, Serinc5, Shisa4, Sipa1l1, Slc16a1, Slc17a6, Slc17a7, Slc27a4, Slc30a3, Slc4a10, Slc6a17, Slc7a8, Slc9a3r1, Smarca5, Snap25, Snrpd3, Snta1, Snx27, Spr, Ssr4, Stx1b, Stxbp5l, Sucla2, Sv2b, Sv2c, Syngr1, Syt1, Syt2, Syt3, Syt7, Them6, Thy1, Tmem63b, Tmx1, Tnr, Tppp, Tspan2, Tspan9, Ttyh2, Tuba8, Tubb2b, Tubb3, Uba2, Ube2n, Usp10, Usp14, Vamp1, Vamp2, Vcan, Vdac2, Vkorc1l1, Vps52, Vsnl1, Wfs1, Xpo6, Ybx1
C1qa, C1qb, C1qc, Ctsb, P2ry12, Sirpa
Acsl6, Aldh1l1, Aqp4, Atp1b2, Bcan, Dclk1, Entpd2, GFAP, Gja1, Gpr37l1, Gria2, Mfge8, Slc4a4, Slc6a1, Slc6a11, Slc7a10, Tlcd1, Trim9
Apod, Gpr37, Lpar1, Mbp, Mog, Plp1, Tspan2, Tubb4a
*Common EV proteins*
CD9, CD63, CD81, CD82
Anxa1, Anxa2, Anxa3, Anxa5, Anxa6, Anxa7, Anxa11
Rab1A, Rab1b, Rab2a, Rab2b, Rab3a, Rab3b, Rab3d, Rab4a, Rab4b, Rab5a, Rab5b, Rab5c, Rab6a, Rab6b, Rab7a, Rab8a, Rab8b, Rab10, Rab11b, Rab12, Rab14, Rab18, Rab21, Rab23, Rab35
Hsd17b12, Hsd17b4, Hsp90aa1, Hsp90ab1, Hspa12a, Hspa2, Hspa4, Hspa4l, Hspa5, Hspa8, Hspb1, Hspd1, Hsph1
EEA1, Vps4b, Vta1
*Absence of non-EV proteins*
Bcl-2, GM130, Nup, Cyc1
The complete MS proteomics data are available via PRIDE with identifier PXD031947

Also, note that the proteomic analysis detected common EV proteins but didn’t identify non-EV related proteins

**Fig. 4:**
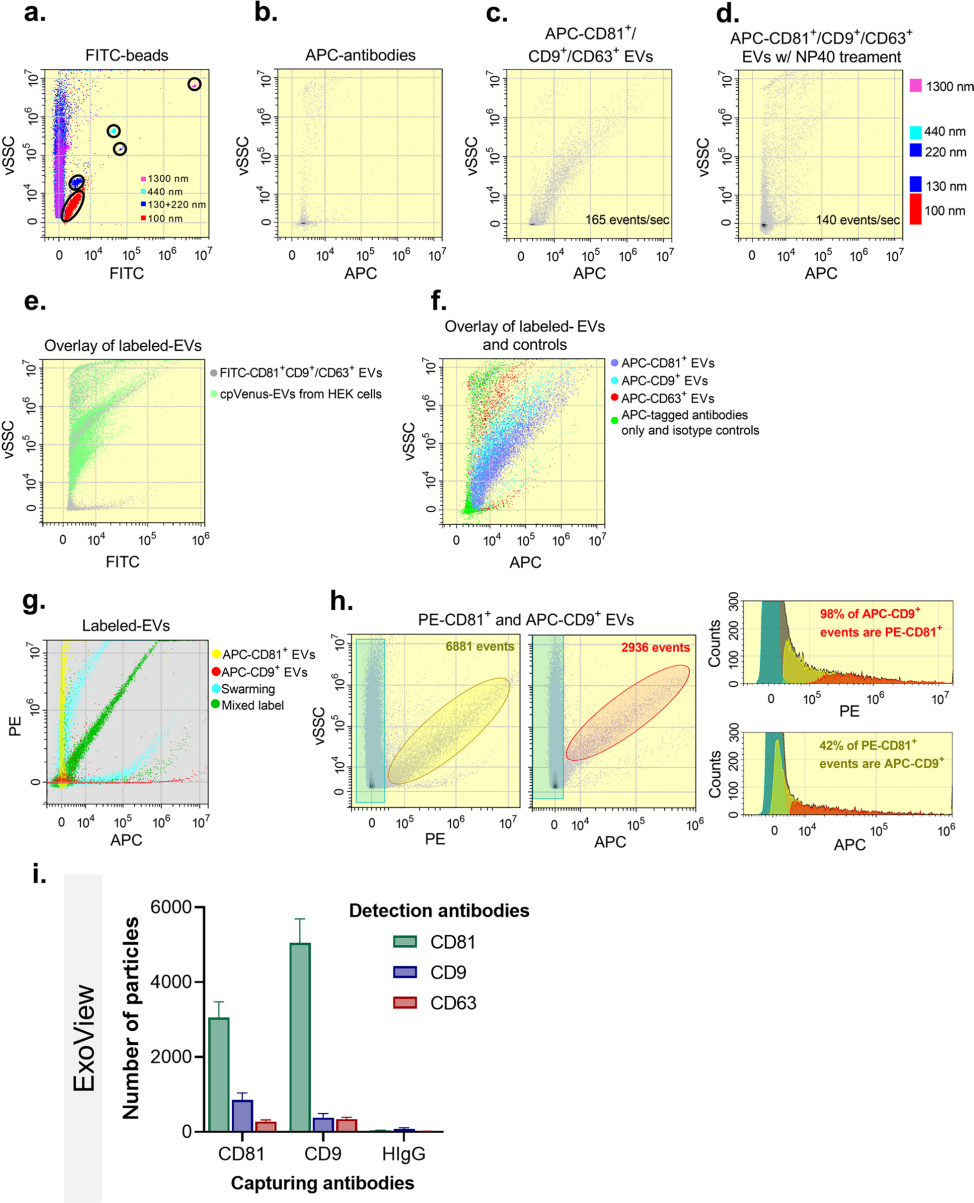
The release method enriches for subpopulation of small EVs. **a** Plot of size-specific events based on FITC-tagged beads (vSSC, size-related measurement). **b** Density and dot plots show the vSSC (size-related measurement) in function of the fluorescent signal representing positive events in samples APC-antibodies alone (**b**). **c**–**e** EVs simultaneously labeled with APC-tagged anti-CD81, -CD9, and/or CD63 antibodies in the absence (**c**) or presence of NP40 detergent (**d**) and overlayed EVs isolated from the conditioned media of cpVenus-HEK cells by ultracentrifugation (**e**). **f** Overlay of EVs individually labeled with the three APC-tagged antibodies and antibodies controls. **g** Representative overlay of PE vs. APC fluorescence signal of individually (PE-CD81, yellow; APC-CD9, red) or simultaneously (green) labeled EVs of the release method, as well as the swarming effect (turquoise). **h** Total number of events of simultaneously labeled EVs as a function of PE or APC fluorescence, respectively (turquoise gate: background; yellow gate: PE^+^ events; red gate: APC^+^ events). Relative abundance of gated events as a function of PE or APC fluorescence within total events (grey). **i** ExoView analysis of the number of positive particles for CD81 or CD9 (N = 3 samples)

In addition, we used high-sensitivity nanoscale flow cytometry analysis to characterize the expression of surface EV markers further [27]. Particle size scatter was detected using a 405 nm (violet) wavelength laser (vSSC), an indicator of particle size that has been shown to improve EV resolution [32]. To control for swarming effects and overestimation, the flow rate was controlled to keep the frequency of events under 200 events/second. Simultaneous labelling with APC-tagged anti-CD9, CD63, and CD81 antibodies resulted in the detection of APC+ events with a vSSC range within that of fluorescent beads within 100–1300 nm size-range (Fig. 4a–c), whereas negative controls showed low electronic noise and no signs of particle detection (antibody-alone [Fig. 4b], water and EVs labeled with isotype controls [Additional file 1: Fig. S5a–c]). To confirm the vesicular nature of the positive events, we treated EV fractions with NP40 detergent (expected to disrupt exosomes), which resulted in diminished APC+ detection (Fig. 4d). In line with their vesicular composition, the isolated EVs showed similar scattering and fluorescent properties as EVs isolated from the conditioned media of cpVenus-expressing HEK293 cells (Fig. 4e). Next, we sought to evaluate the relative abundance of various tetraspanins in individually labelled samples (Fig. 4f, Additional file 1: Fig. S5d–f) in order to identify EV subpopulations as previously described [27], and found that CD9+ and CD81+ EVs were the most abundant. To further explore this finding, we sought to quantify the CD9+/CD81+ EV subpopulation after confirming our capacity to distinguish between single- and double-positive events (Fig. 4g). Analysis of simultaneously labeled EVs (Fig. 4h) showed that 95.05 ± 4.90% of APC-CD9+ events are PE-CD81+, whereas 32.31 ± 18.82% of PE-CD81+ events are APC-CD9+. These results concur with previous observations indicating that CD81 is expressed by most smaller EVs, whereas CD9 and CD63 are present in EV subpopulations of a wider range of sizes [27], further suggesting that EVs isolated by the release method were enriched for smaller EVs. Finally, ExoView analysis (NanoView Biosciences) verified the abundance of CD81 and CD9 in EVs isolated with the release method (Fig. 4i), providing further support to the findings of our high-sensitivity nanoscale flow cytometry analysis.

### Significance of release method under chronic stress conditions and drug-induced modulation of EV biogenesis

We next monitored the significance of release method under pharmacological regulation of EVs biogenesis and secretion as well as after exposure to chronic stress—prolonged stressful life events are suggested to increase susceptibility to neurological disorders such as Alzheimer’s disease and depression while chronic stress is casually associated with memory and mood deficits [33].

**Fig. 5:**
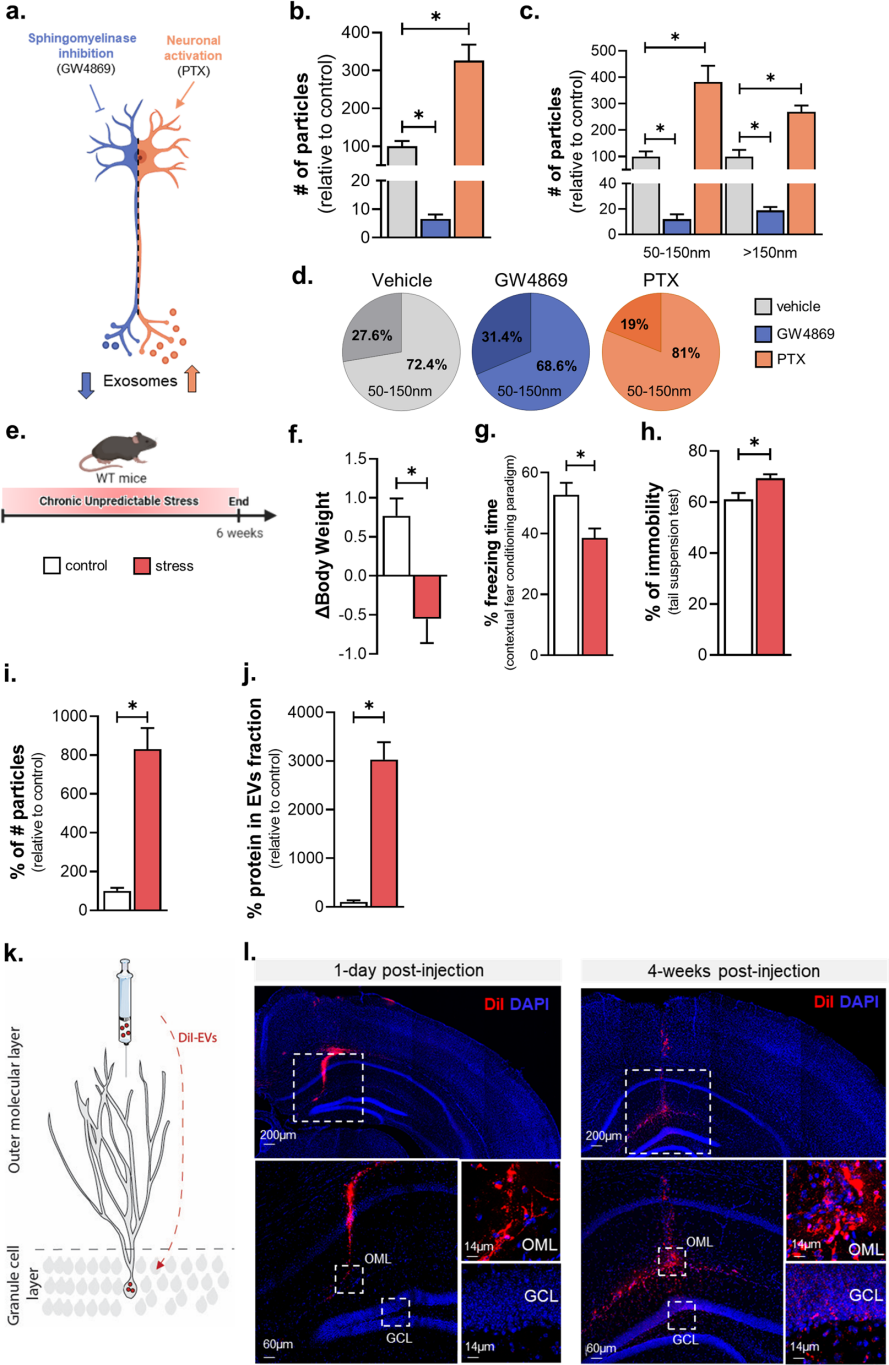
The release method detects alterations of EV biogenesis and isolates intact EVs that propagate in the brain. **a** Schematic representation of the experiment of inhibition or activation of exosome biogenesis/secretion by GW4869, an inhibitor of neutral sphingomyelinase, and Picrotoxin (PTX), an inhibitor of GABA_A_ receptors that increases excitatory neuronal firing. **b–d** Brain tissue was incubated with GW4869 or PTX. Nanoparticle tracking analysis (NTA) of the number of particles (**b**), particles divided by size (**c**), and relative abundance of smaller (50-150nm) and bigger (>150nm) EVs (**d**) (N_*vehicle*_ = 5, N_*GW4869*_ = 3 N_*PTX*_ = 3). **e** Schematic representation of the stress protocol timeline. **f** Body weight change as a measurement of stress efficacy. **g** Percentage of freezing behavior in the Contextual fear conditioning test. **h** Percentage of immobility in the Tail suspension test. **i** Nanoparticle tracking analysis (NTA) of the number of particles by tissue weight detected in stressed and control animals. **j** microBCA analysis of the amount of protein by tissue weight in stressed and control animals (**k**) (behaviour: N_*control*_ = 10, N_*stress*_ = 10; EVs: N_*control*_ = 4, N_*stress*_ = 4). **k** Schematic representation of the DiI-labeled EVs injected into the outer molecular layer (OML) of the dentate gyrus of mouse hippocampus and their spreading to the granule cell layer (GCL). **l** Confocal images showing Dil-labeled EVs (red) at the site of injection (OML) at 1-day post-injection and the spread of Dil signal onto the GCL of dentate gyrus 4 weeks later (N_*1day*_ = 8, N_*4weeks*_ = 8). All data shown represent mean ± SEM *p < 0.05 by two-tailed Welch’s t-test or one-way ANOVA

For ex vivo pharmacological regulation of EVs, we targeted cellular processes involved or related to the biogenesis/secretion of small EVs (e.g., exosomes) by triggering (i) the inhibition of endosomal EVs biogenesis by tissue treatment with GW4869, an inhibitor of the neutral sphingomyelinase/ceramide synthesis pathway, and (ii) the enhancement of EV release by increasing neuronal activity with picrotoxin (PTX) treatment, an inhibitor of GABA_A_ receptors that is known to lead to increased neuronal activity [34] (Fig. 5a). As shown in Fig. 5b, c, GW4869 treatment led to a significant reduction of recovered EVs while the PTX treatment increased the number of total EVs isolated, but it did not affect the overall abundance of two size-based populations compared to control conditions (Fig. 5d) (F_total # of particles_ (2,8) = 2.282, *p* < 0.0001; Tukey’s post-hoc: *p* < 0.05; F_50–150 nm_(2,8) = 2.439, *p* < 0.05; Tukey’s post-hoc: *p* < 0.05; F_>150 nm_(2,8) = 0.8467, *p* < 0.05; Tukey’s post-hoc: *p* < 0.05). Note that none of the two conditions, that affect EV biogenesis and/or secretion, significantly changed the size of EVs (Additional file 1: Fig. S6a). However, there is a tendency for an increased EVs size under GW4869 treatment depicted by both mean particle size (Additional file 1: Fig. S6a) and percentage of > 150 nm particle subpopulation (Fig. 5d), which may suggest a more prominent role for neutral sphyngomielinase on the biogenesis of smaller EVs (50–150 nm) compared to bigger ones (> 150 nm).

For the in vivo experiments, we exposed wildtype mice to chronic stress, a known risk factor for brain pathologies such as depression and Alzheimer’s disease which is known to affect both cognition and mood [33]. We used the 6-weeks-long protocol of Chronic Unpredictable Stress (CUS) [13]. As shown in Fig. 5e, f, animals under chronic stress exhibited reduced body weight in contrast to control (unstressed) animals supporting the efficiency of our stress protocol. Furthermore, stressed animals displayed reduced freezing time in contextual fear conditioning text compared to control animals indicating memory impairment (Fig. 5g; t-test with Welch’s correction, t(2.795) = 17.19, *p* = 0.0123). In addition, stressed animals also expressed depressive-like behavior exhibiting increased immobility time in Tail suspension test (Fig. 5h; t-test with Welch’s correction, t(2.756) = 15.25, *p* = 0.0145). In parallel, we found that chronic stress triggers an increase in the number of EVs of brain tissue which was accompanied by a similar increase of EV protein content monitored by microBCA; however, no stress-driven change in the mean size of particles is detected (Fig. 5i–j), suggesting a potential role for sEVs in the cellular responses of the brain tissue under stressful conditions.

Next, we monitored the integrity of EVs isolated by the release method by monitoring their in vivo internalization and movement/spreading in the brain of live animal as previously described [35]. For that purpose, we labelled EVs of the release method using DiI dye (dioctadecyl-3,3,3′,3′-Tetramethylindocarbocyanine). Then, we stereotaxically injected Dil-labelled EVs diluted in sterile PBS into the Outer Molecular Layer (OML) of the dentate gyrus (DG), where the dendritic tree of the granule cells is located (Fig. 5k). Next, we monitored the potential movement of Dil signal in the granular cell layer (GCL) of DG. As shown in Fig. 5l, on day 1, the signal of DiI-labeled EVs (red) was detected at the place of injection (OML) whereas no Dil signal was found in GCL. Importantly, 4-weeks post-injection, Dil signal was detected in GCL, indicative of potential movement of Dil-labelled EVs as previously described [35]. Note that when Dil dye alone (in absence of EVs) was injected in the OML, no such movement of Dil signal in GCL was detected at either 1-day or 4-week post-injection timepoint (Additional file 1: Fig. S6d,e), suggesting an EV-dependent movement of the Dil signal from OML to GCL of mouse DG. Moreover, to verify that the DiI dye itself did not form mycelles during the EV isolation process, we also injected DiI-PBS that underwent the full protocol of the release method. Injection with either Dil processed via EV isolation protocol or PBS did not result in Dil signal in OML or GCL (Additional file 1: Fig. S6b, c).

## Discussion

Isolation and characterization of subpopulations of pure and physiologically relevant small EVs (sEVs) from the brain is of paramount importance for advancing our understanding of EV biology and its potential use for disease diagnosis, prognosis, and treatment [9, 36, 37]. Over the past 30 years, EVs have been successfully isolated from cell culture media and body fluids (e.g., CSF, blood, urine, sperm, breast milk), with specific precautions taken into account to decrease the interference from non-EVs structures [38]. The isolation of EVs directly from the brain is an even more challenging procedure, as tissue is a complex structure, and EVs must be liberated from the extracellular matrix. To accomplish this, brain tissue suffers an initial mechanical disruption and subsequent enzymatic digestion to disrupt the network that composes the extracellular matrix. During this disruptive procedure, artifacts, such as synaptosome-like vesicles formation, membrane damage, or contamination with intracellular vesicles may occur. Indeed, in the past, it was shown that papain digestion, and other enzymes, alter the composition of the plasma membrane [39, 40]. Indeed, this could explain why the proteome of EVs yield of the release method (but not the standard one which is based on tissue digestion by papain) is particularly enriched in membrane and extracellular region categories. While the release method includes many different steps compared to the standard method/protocol of EVs isolation (including differential tissue processing), the main conceptual difference between the two methods relies on the spontaneous release of EVs from the cells of the origin tissue. Thus, the release method is expected to overcome the drawbacks of previous methods for the isolation of brain EVs involving tissue digestion as the release method isolates EVs from the culture medium which implies that EVs are released from the recipient cell to the extracellular space, including brain interstitial fluid, before reaching the culture medium. Without excluding the possibility that part of the total population of EVs remains in the brain interstitial fluid, the multiscale analysis of the current studies suggests that the release method isolates a sEV-enriched yield with EVs being structurally, biochemically, and functionally intact. Thus, the released method constitutes a novel tool for detailed “mapping” of sEVs released directly from brain cells, which may enable the discovery and validation of brain disease biomarkers from peripherally-collected EVs (e.g., blood, cerebrospinal fluid, or other biofluids) and help identify cell-specific markers of brain EV subpopulations (i.e., neuronal, astrocytic, oligodendrocytic and microglial-derived EVs).

Moreover, this method offers a great opportunity for the study of brain EV biogenesis and modulation ex vivo. Indeed, as message conveyers, EVs have been suggested to take part in the initiation, progression, and treatment resistance of several diseases. For instance, EVs were shown to contribute to metastatic niche formation [41–43] and even suppress immune cells involved in cancer cells surveillance. In addition, EVs were shown to confer resistance to chemotherapy drugs [44]. Moreover, in several neurodegenerative disorders, EVs have been suggested as propagators of pathological processes, contributing to brain function deterioration over time [35]. For instance, exosomes are suggested to be involved in Tau propagation between cells and brain areas in Alzheimer’s disease brain pathology. Consistent with suggestions that lifetime stress may be a clinically-relevant precipitant of AD and depressive pathologies, we previously showed that chronic stress and high levels of the main stress hormones, glucocorticoids, may trigger neuronal activation leading to accumulation of hyperphosphorylated Tau species in neurons by dysregulation of major degradative pathways such as the endolysosomal pathway and autophagy [45, 46]; both pathways are involved in sEV (e.g. exosome) biogenesis and secretion. Together with the current findings showing that chronic stress increases the sEV secretion, the release method provides a novel window of opportunity—via the spontaneous EVs release—that may contribute to a better understanding of cellular response and (mal)function when the brain is under high stress load. As brain sEVs (e.g. exosomes) seem a promising biomarker “tool”, future studies should explore the potential role of sEVs and probably exosomes in the etiopathogenesis and diagnosis of stress-related brain pathologies.

Thus, there is a high demand for an EV-based method that reliably evaluates the efficacy of predisposing factors for brain pathology as well as the novel compounds that target EV-related processes to ameliorate pathology. Furthermore, EVs low immunogenicity and their ability to cross the blood–brain barrier make them perfect candidates for new drug delivery systems. Indeed, as they carry cell-specific markers, engineered EVs are primed to become the next generation of cell-specific drug delivery systems overcoming considerable side-effects associated with other drugs. In addition, one needs to consider the source of EVs as it can affect membrane composition and consequently change their homing capacity and biological function [47]. Thus, spontaneously-released EVs collected from an individual patient can open new therapeutic avenues for various brain disorders in the era of Precision medicine and personalized treatment.

## Supplementary Information

**Additional file 1.** Supplementary figures, material and methods.

## Data Availability

The complete MS proteomics data are available via PRIDE with identifier PXD031947.
