# Supplementary material for: A novel isolation method for spontaneously released extracellular vesicles from brain tissue and its implications for stress-driven brain pathology
Source: Cell Commun Signal. 2023 Feb 13;21:35. doi: 10.1186/s12964-023-01045-z (PMC9926669; doi:10.1186/s12964-023-01045-z)

Supplementary Material

Supplementary figure 1. Cryo-EM comparison of EVs isolated by both methods. a) Additional cryo-EM micrographs of brain EVs isolated by the standard and release methods; EVs are highlighted by black arrowheads; multi-lamellar highlighted by white arrows; scale bar = 100nm. **b**) Correlation between protein levels and the number of particles in the standard (left) and release (right) methods; the color bar represents the tissue weight used to isolate the EV yield. $N_{standard}$=8, $N_{release}$=7.

Supplementary figure 2. EVs from different brain areas are isolated by the release method. a-d) EVs isolated by the release method from the entorhinal cortex, hippocampus, and cortex. NTA of particle mean size (**a**), the size distribution of EVs (**b**), and percentage of small (grey area; 50-150nm) and bigger EVs (>150nm) (**c**). **d**) Correlation between the number of particles and tissue weight; note that the higher the tissue weight, the more particles (EVs) are isolated. **e**) Correlation between the size of particles and tissue weight; note that tissue weight does not affect the size of isolated EVs; ($N_{entorhinal}$=4, $N_{hippocampus}$=4, N_{cortex}=8).

Supplementary figure 3. **Quality control of brain tissue at the end of the incubation period of the release method. a-b**) Trypan blue-based assessment of the total number of cells and percentage of alive cells. **c-e**) Representative immunoblot of Cyt C and NSE (**c**) and assessment of the impact of the incubation period and H_2O_2 on the relative abundance of these proteins - Cytochrome C (CytC) (**d**) and Neuron-specific enolase (NSE) (**e**). Numerical data are presented as mean ± SEM. N=9 per condition. *p<0.05 by one-way ANOVA.

Supplementary figure 4. GO analysis of the proteome of EVs of the release method. The size of the circle (small to big) represents the enrichment within the sample (% of detected proteins of the GO category vs. the total number of proteins detected in the EVs sample), while the color bar - purple (low) to yellow (high) - represents the enrichment within each GO category (ratio of the detected proteins of the specific GO category in the EV sample vs. the total proteins of the GO category.

Supplementary figure 5. Characterization of EVs from release method with high-sensitivity nanoscale flow cytometry analysis. EVs from the release method (3 samples) were subjected to fluorescent antibody labeling and flow cytometry analysis. **a-c**). Density and dot plots show the vSSC (size-related measurement) in the function of the fluorescent signal representing positive events in samples containing only water (**a**), EVs incubated with APC-tagged isotype controls (**b, c**). **d-f**) EVs individually labelled with APC-tagged anti-CD81, -CD9, and/or CD63 antibodies.

Supplementary figure 6. EVs mean size after EV biogenesis manipulation and negative controls of the experiment of brain injection of DiI-labeled EVs. a) NTA-based mean size of EVs following GW4869 or PTX treatment. $N_{vehicle}$=5, N_{GW4869}=3, N_{PTX}=3. **b**) No DiI signal (red) was detected in the OML or GCL upon PBS injection**. c)** No DiI signal was detected upon injection of EVs-free DiI (DiI processed through the release method in the absence of EVs [non-bound-to-EVs DiI]).

This may indicate that DiI is not precipitated by the release method protocol in the absence of EVs. **d-e**) DiI dye alone injected in the OML remains at the place of injection (OML – see red signal) either 1 day or 4 weeks post-injection (N_{PBS}=4, N_{DiI}=4, N_{1day}=8, N_{4weeks}=8). All numerical data are shown as mean ± SEM *p< 0.005.

Supplementary Material and Methods

Quality control of brain tissue. At the end of the respective incubation period (0h, 16h, or 40h) or H_2O_2 treatment (16h, 1%), brain samples were immediately digested with papain to assess the cell viability as previously described[27]. Briefly, tissue was incubated for 30min in a shaking 30ºC-water bath in a tube containing previously activated papain [12mg of papain (Worthington, #3119) in Hibernate-A (A1247501, Gibco™) at 37ºC for 20min). After transferring the tissue to a new falcon with 2mL Hibernate-A, the tissue was dissociated with a siliconized 9-in Pasteur pipette (approximately ten times in 45sec without air bubbles). Pieces were allowed to settle for 1min, and the supernatant was transferred to a new 15mL falcon. New hibernate-A was added to the pellet, and dissociation was performed again – this process was repeated one more time. From the tube with cells in suspension, 100ul were collected and added to 100ul of 0.4% trypan blue (15250061, Gibco™). Then, this solution was added to a glass hemocytometer and observed under the microscope with a 10x objective. Unstained and stained cells were counted for viability estimation. For western blot analysis of the brain tissue at the end of the incubation period (0h, 16h, or 40h) or H_2O_2 treatment, brain tissue was homogenized by RIPA buffer (50mM Tris-HCl, 2mM EDTA, 250mM NaCl, 10% glycerol) with protease and phosphatase inhibitors. The homogenate was centrifuged at 14.000rpm for 15min at 4ºC. The supernatant was collected and stored at -80ºC. After Laemmli buffer addition and heat-denaturation (95ºC, 10min), SDS-PAGE electrophoresis and semi-dry transfer onto nitrocellulose membranes were performed (Trans-Blot Turbo blotting system, BIORAD). Then, membranes were blocked with 5% non-fat dry milk in TBS-T buffer and then incubated with the following antibodies in 2.5% non-fat dry milk in TBS-T buffer: cytochrome C (1:1000, ab13575, ABCAM) and neuron-specific enolase (1:1000, MAB324, Millipore). After overnight incubation (4ºC), membranes were incubated with appropriate secondary antibody (1:10000, HRP Goat anti-Mouse or anti-rabbit, #1706516, #1706515, BioRad, respectively) for 2h at RT, and antigen signal was revealed using Sapphire Biomolecular Imager (Azure Biosystems, Dublin, CA) and AzureSpot software (Azure Biosystems, Dublin, CA).

Supplementary figure 1.

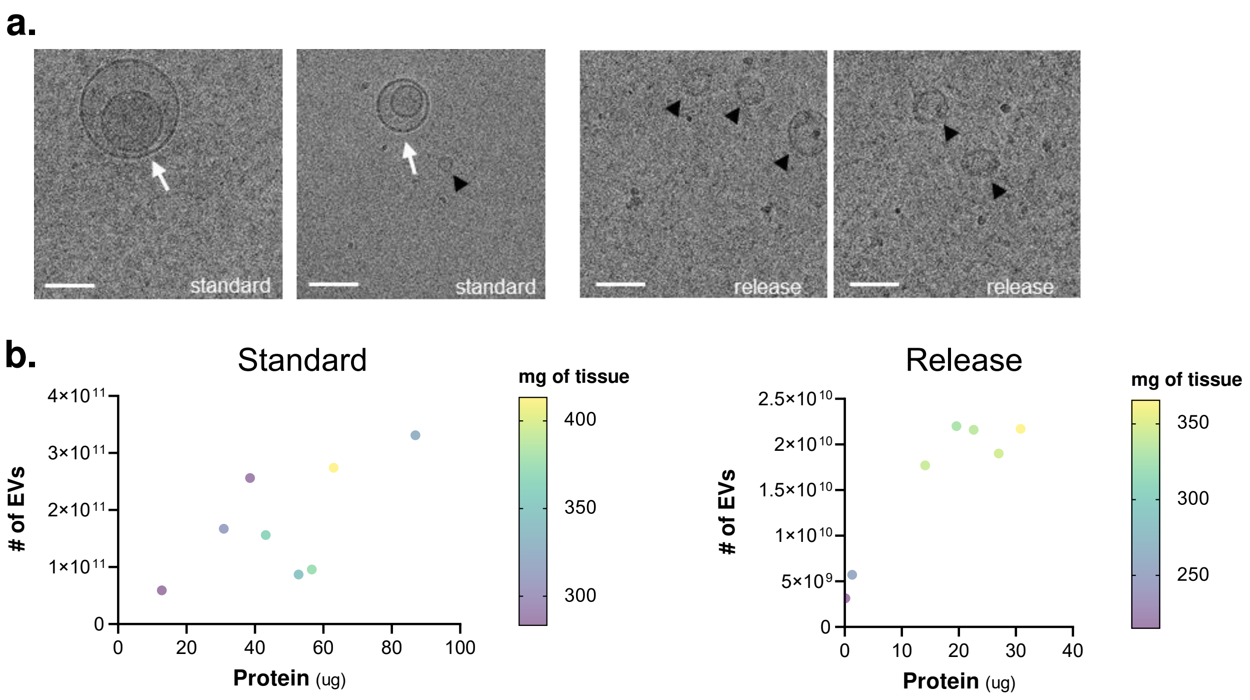

Supplementary figure 2.

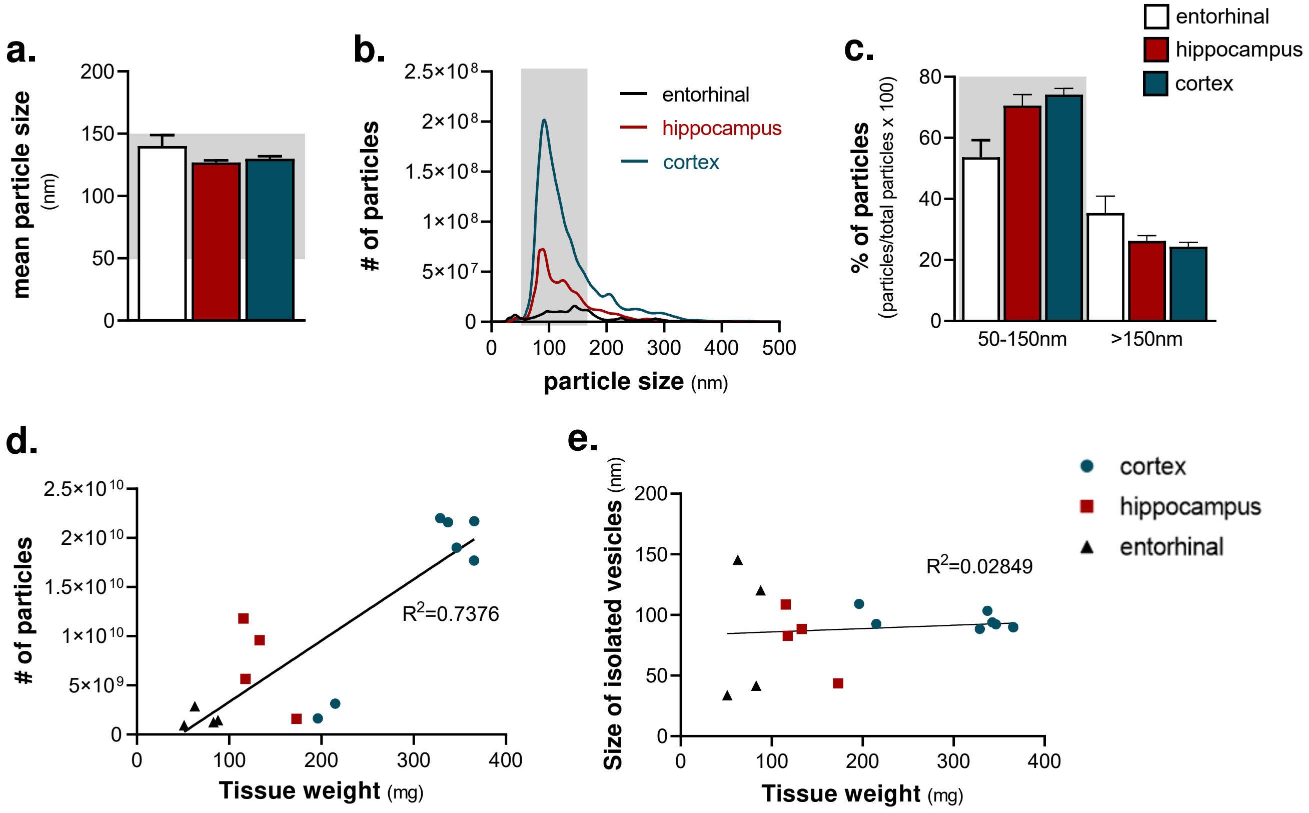

Supplementary figure 3.

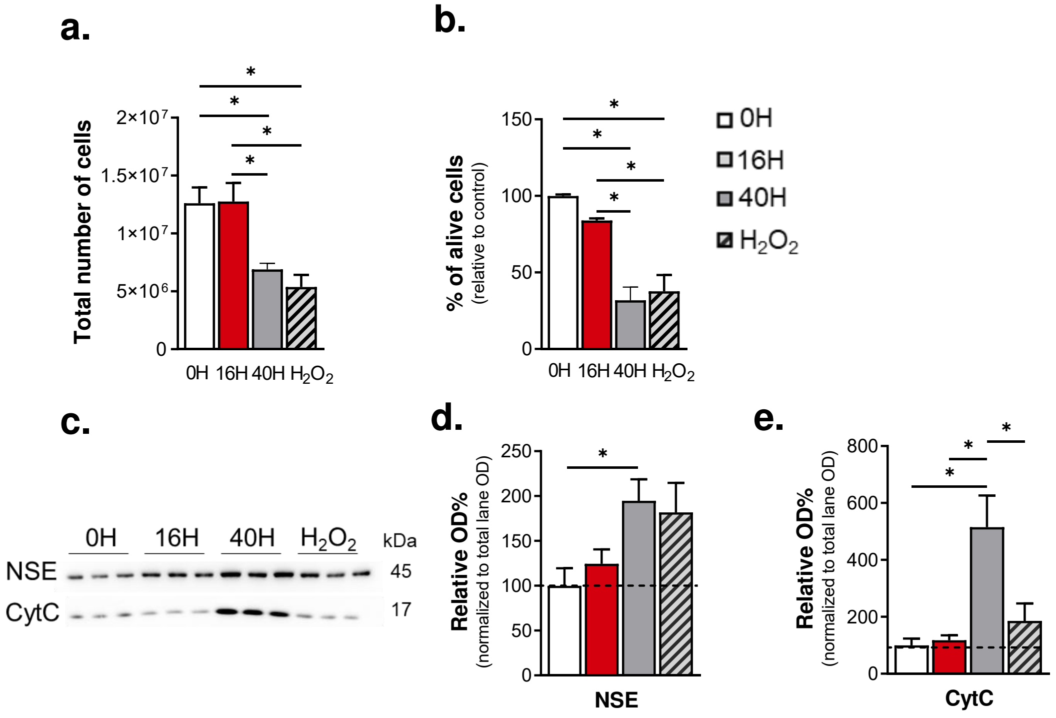

Supplementary Figure 4

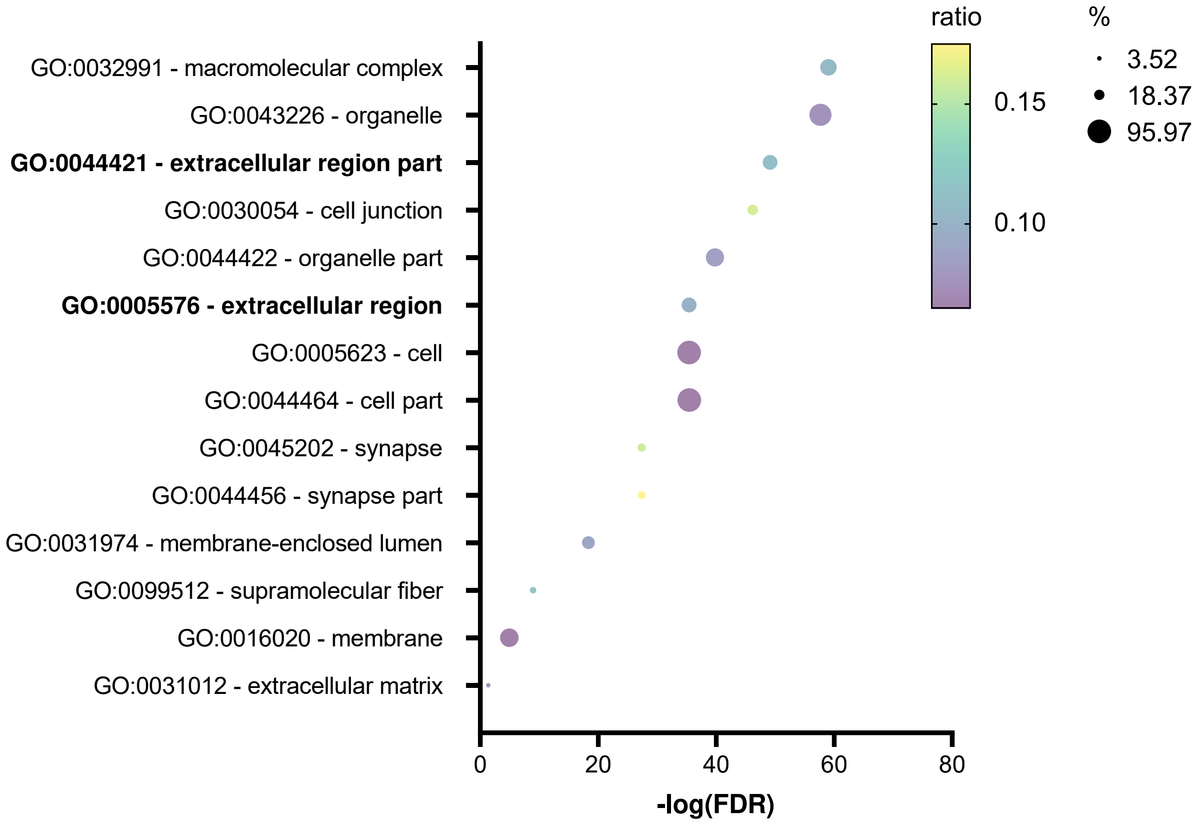

Supplementary Figure 5

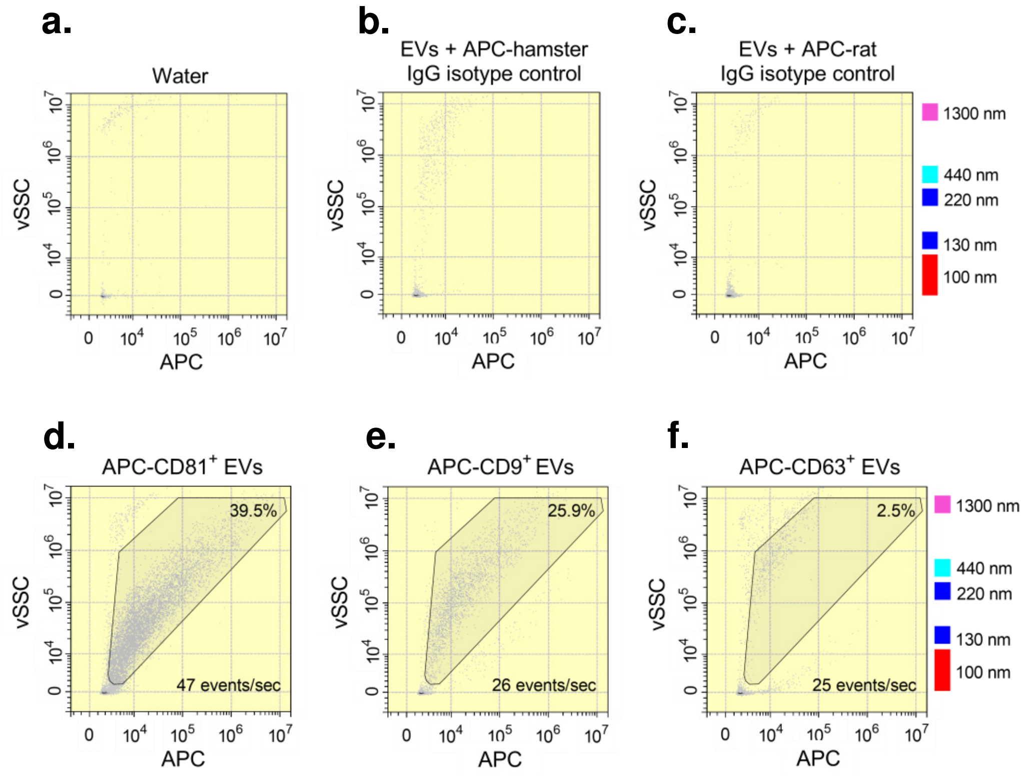

Supplementary figure 6.

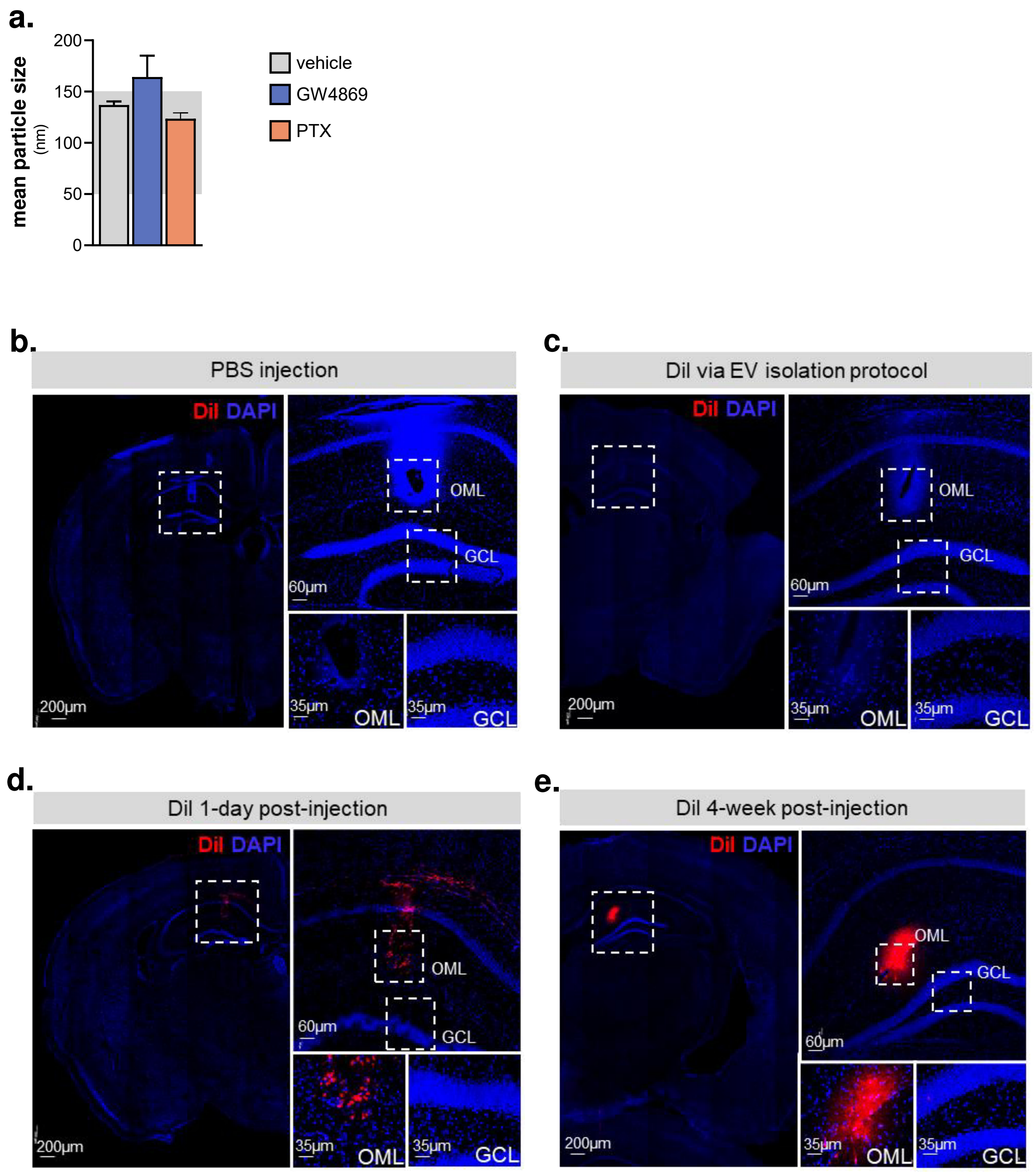

Supplement: Supplementary file 2 — Additional file 1. Supplementary figures, material and methods. [file 12964_2023_1045_MOESM2_ESM.pdf]
